# Fiber and Polymer Composites: Processing, Simulation, Properties and Applications II

**DOI:** 10.3390/polym16243486

**Published:** 2024-12-14

**Authors:** Zina Vuluga

**Affiliations:** National Institute for Research and Development in Chemistry and Petrochemistry—ICECHIM, 202 Splaiul Independentei, 060021 Bucharest, Romania; zvuluga@icechim.ro

Compliance with EU legislation on the efficient use of fossil fuels and the reduction in emissions and environmental impact has led many sectors of industry to become interested in obtaining and using more sustainable polymer composites with improved lifetime, in the recovery and recycling of materials at the end of their life cycle, and in the use of renewable natural resources. Polymer composites contain a polymer matrix, thermoplastic, or thermoset, synthetic and/or natural and synthetic and/or natural fillers as reinforcing agents. For the construction and automotive industry, the most important consumer sectors of polymers and polymer composites, fiber-reinforced polymer composites have become key materials. These fiber-reinforced polymer composite materials are used as an alternative to metals due to their advantages such as light weight, high stiffness, corrosion resistance, flame resistance, durability, flexibility, functionality, and formability into parts with complicated designs [1].

This Special Issue “Fiber and Polymer Composites: Processing, Simulation, Properties and Applications II” contains 20 original research articles and 1 review which contribute to the progress of industry in particular, in its attempt to replace metals with reinforced polymer composites, but also in other fields (e.g., medicine).

Fibers can be natural (e.g., plant fibers), synthetic (e.g., carbon fibers, glass fibers, and polymer fibers), and artificial (e.g., collagen fibers). Fibers are generally classified into short or discontinuous fibers and long or continuous fibers (from Wikipedia).

The mechanical performance of fiber-reinforced composites depends on a series of factors, starting from the method of production and the type and properties of the fibers and continuing with the manufacturing processes, pretreatment processes, and fiber content.

Glass fibers (GFs) are commonly used as reinforcing agents in thermoplastic or thermosetting polymers, due to the improved rigidity and dimensional stability properties it provides them [2,3,4]. To obtain improved properties required by various applications, it is necessary to treat the fiber surface to achieve strong adhesion at the polymer matrix–fiber interface. A good interface adhesion in a GF-reinforced polyester composite was obtained by treating unidirectional GF with oxygen plasma followed by plasma coating [3]. The interface can be controlled by adding maleic anhydride-grafted polymer and thermoplastic elastomers to obtain reinforced polymer composites with a good stiffness–toughness balance [1,4]. The specific properties required by certain fields of application are achieved by using high GF concentrations (30–50 wt.%), which creates great problems both in obtaining reinforced polymer composites and further in the manufacture of parts. Therefore, numerous attempts have been made to reduce the fiberglass content and to use non-abrasive, high-performance, and cheaper reinforcing agents. Thus, in polypropylene (PP) reinforced with 30 wt.% GF, a commercial product, 5 wt.% by weight GF was replaced with fly ash from the thermal power plant and, by melt processing, a composite with improved (nano)mechanical properties was obtained. The new composite material is an efficient and low-cost alternative to PP reinforced with 30 wt.% GF for obtaining exterior automotive parts [4].

Often, to obtain special properties and maintain them under extreme conditions (i.e., low temperature and/or ice coating), carbon fibers (CFs) are preferred, although they are more expensive but twice as durable and 30% lighter than GF. Studies conducted under various electric currents on CF/epoxy composites have proven their effectiveness in deicing [5]. Sizing of the reinforcing fibers is another method to increase the interfacial adhesion between the fiber and matrix. Therefore, by applying two commercially available sizing formulations to the surface of CF, the surface defects of the fibers were covered and, consequently, a 6% increase in the ultimate tensile and interfacial shear strength of reinforced epoxy resin composites was achieved [6].

To obtain composite materials with improved and durable mechanical properties, it is very important to take into account the geometry and mechanical properties of the fibers selected to be used [7]. In some applications in fields such as (bio)medicine and electronics, special importance is given to polymer fibers. Electrospinning is the most widely used method for obtaining micro- and nanofibers from synthetic and natural polymers, with high porosity and a high surface-to-volume ratio, due to its ease of application and wide range of options for parameter optimization. By optimizing the electrospinning parameters and the concentration of a low-molecular-weight polyvinylpyrrolidone (PVP) solution, uniform fibers could be obtained [8]. The surface area per unit volume and the porosity of the fibers depend on the properties of the solution/emulsion that is electrospun and, on the other hand, on the electrospinning conditions, respectively. Digital image analysis (DIA) has started to be increasingly used for the study of the porosity of materials. Thus, an empirical mathematical expression was proposed for predicting the digital porosity of electrospun nanofiber veils from polyvinyl alcohol emulsion and olive and orange oils [9]. However, because of low productivity and costs, attempts have been made to find a simpler, cheaper, and high-yield alternative. Thus, using the centrifugal method, it was possible to obtain a fibrous structure without defects, with easily aligned microfibers, from PVP, a fiber-forming polymer, and chlorzoxazone, a centrally acting muscle relaxant and sedative. These chlorzoxazone-containing microfibers can be used as drugs with controlled release of the active substance [10].

To reduce the problems of environmental pollution and resource scarcity, much research has been directed towards replacing synthetic polymers with biopolymers and biodegradable polymers and obtaining durable and high-performance composite materials. Thus, using a combination of bio-based polyol and bio-based glycerol, a new bio-based thermosetting polyurethane (BIO-PUR), with the mechanical properties and processability required for unidirectional GF reinforcement and the fabrication of structural composites, was developed by resin transfer molding (RTM) [11]. In an effort to shift from fossil resources to renewable resources and to develop composite materials reinforced with high-performance yet cheaper and more environmentally friendly reinforcing agents, researchers have turned to the use of natural fibers. Composites with good stiffness–toughness balance have been obtained by melt processing multiphase systems based on PP, hemp fibers, and thermoplastic elastomers [1]. A strontium-doped collagen fiber and hydroxyapatite composite material was uniformly embedded in a polylactic acid (PLA) solution in 1.4 dioxane, and then a scaffold model with interconnected pores was obtained by 3D printing for bone tissue regeneration [12]. The mechanical performance of plant-fiber-reinforced composites depends on the degree of adhesion at the interface. The interface could be controlled by adding maleated polypropylene to hemp-fiber-reinforced PP [1] or by the combined effect of alkali and silane surface treatment applied to the fibers in PLA composites reinforced with 40 wt.% flax fibers [13]. The mechanical performance and usability of composites reinforced with natural fibers for outdoor applications depend on their resistance to environmental factors such as humidity, ultraviolet radiation, and heat. Therefore, many researchers have studied the aging of these composites by exposing them to both natural conditions (natural aging) and simulated conditions in laboratory climate chambers (artificial aging). Effective prediction models have been developed to evaluate the mechanical resistance of biocomposites after exposure to ultraviolet radiation and/or humidity for different times. An artificial neural network (ANN) model was used to predict the tensile and impact resistance of a PP composite reinforced with 30 wt.% of short flax or wood pine fibers [14]. The natural fiber category also includes basalt fibers (BFs), which are not biodegradable but have a higher tensile strength than E-glass fiber, a higher fracture load than carbon fibers, and good resistance to chemical impact after impact load. The tensile and shear behavior of bio-based epoxy resin reinforced with basalt fiber was investigated with digital image correlation and acoustic emission monitoring [15].

To reduce the environmental impact of plastic waste, in addition to using biodegradable materials, the recycling process is considered a more energy- and resource-efficient solution. For example, by recycling high-density polyethylene from milk bottles and reinforcing it with 20 wt.% of dried pineapple leaf fibers, composites with a 162% increase in flexural strength and a 204% increase in modulus were obtained [16]. Another efficient recycling solution is the use of agricultural waste, which has the advantage of being abundant, natural, and biodegradable, but, without their efficient recycling, it would have a substantial impact on the environment. New composites based on wasted date palm surface fibers and pineapple leaf fibers, bound together by means of a wood adhesive as a binder, have been developed. The new materials may be a promising alternative to synthetic and petrochemical materials used as thermal insulation and sound absorption materials in construction [17]. The possibility of the valorization of corn stalks to obtain papermaking fibers, hemicelluloses, and lignin by sequential alkaline fractionation was studied [18].

The performance and durability of various components made from reinforced polymer composites are strongly influenced by the processing parameters, as well as the properties of materials used as bonding agents between two separate items, such as adhesives. Various simulation methods combined with experiments have been used to optimize adhesive performance and mitigate defects that occur in parts due to a non-uniform temperature distribution, for example, during the thermoforming process, and due to the occurrence of internal stresses. By using the finite element model combined with experiments, it was possible to simulate the deterioration of the two-component epoxy resin adhesive used in the manufacture of polyurethane (skin and non-woven fabric) automotive interior components as a result of a non-uniform temperature distribution and stress concentration [19]. Also, a design of experiments (DOE) was used to study the effect of holding time and pressure on the thermoforming behavior and tensile mechanical properties of unidirectional BF-reinforced polyamide 6 laminates. Through a regression model, the properties of the laminate were predicted and validated [20]. In other situations, the performance of the finished materials depends on the properties of the background materials, as in the case of textile fabrics made of polymer fibers applied to various substrates. The influence of some support materials (a standard aluminum foil and a standard black foil) on the cooling performance of semi-transparent and opaque textiles (a standard polyester fabric and a standard polyamide fabric) was studied by applying simplified simulation models. Silicone-coated textiles were studied compared to uncoated textiles [21].
